# Untargeted Metabolomics Coupled With Machine Learning Unravels Crop‐Specific Versus Generalized Effects of Biostimulants in Cucumber, Pepper, and Tomato

**DOI:** 10.1111/ppl.70904

**Published:** 2026-04-28

**Authors:** Hajar Salehi, Pascual García‐Pérez, Luigi Lucini

**Affiliations:** ^1^ Department for Sustainable Food Process, CRAST Research Centre Università Cattolica del Sacro Cuore Piacenza Italy; ^2^ Department of Food Technology, Nutrition and Food Science, Veterinary Faculty University of Murcia, Regional Campus of International Excellence “Campus Mare Nostrum” Murcia Spain; ^3^ Institute of Bioimaging and Complex Biological Systems (IBSBC) National Research Council (CNR) Milan Italy

**Keywords:** biomarker discovery, DIABLO data integration, integrative analysis, intercrop variability, sustainable agriculture

## Abstract

In recent years, agricultural practices have shifted toward sustainability, aiming to reduce the use of agrochemicals and rely more on bio‐based solutions. However, the effectiveness of these latter suffers from inconsistency. Understanding how different crops respond to biostimulants, instead of referring to a specific trial or crop, is a challenge in this field. This study attempts to identify common metabolite signatures associated with different commercial biostimulants across three crops, moving from trial‐specific to more generalized effects in horticultural crops. To this aim, advanced metabolomics data integration and supervised statistical methods have been used. Advanced multivariate analyses included analysis of variance (ANOVA)‐multiblock orthogonal partial least squares (AMOPLS), and Data Integration Analysis for Biomarker discovery using Latent variable approaches for Omics studies (DIABLO). HCA and AMOPLS revealed differences in metabolic profiles among the biostimulant treatments, while confirming crop‐specific responses. Data integration indicated that three metabolites, betaine, *N*‐caffeoylputrescine, and 2‐amino‐4‐hydroxypyrimidine‐5‐carboxylic acid, were consistently modulated across all three crops treated by the multi‐component biostimulant containing osmolytes and zeatin. Notably, these metabolites are known to be involved in plant growth and adaptation to different abiotic stresses. Overall, the applied analytical approach enabled the identification of putative markers within complex metabolic datasets that included different crop species. The use of independent validation methods increases confidence in these markers and supports the integration of complementary datasets in biostimulant studies.

## 1 Introduction

Despite the widespread and long‐standing reliance on synthetic agrochemicals to address environmental challenges, the European Green Deal, through the Farm‐to‐Fork Strategy, sets a specific target to make Europe the first climate‐neutral continent by 2050. This strategy highlights the role of organic farming in reducing the use of agrochemicals by 20% and, as a result, in obtaining a healthier, eco‐friendly, and resilient food system (Council 2022). The use of biostimulants, which are defined as natural substances derived from various sources, is one of the agricultural practices that stepped forward (Ruzzi et al. 2024; Shahrajabian et al. 2021). Biostimulants have been reported to improve plant production and quality by enhancing nutrient use efficacy and boosting stress tolerance (Zulfiqar et al. 2024). Nonetheless, inconsistent findings can be observed across different studies. Results from a recent meta‐analysis, including 180 studies that were conducted in open field conditions, revealed a complex correlation between the efficiency of biostimulants and various factors, including soil characteristics, plant species, application methods, environmental conditions, biostimulant composition, plant growth stages, as well as interactions with other inputs such as fertilizers and pesticides. For instance, the results showed that yield varies from 12.6% to 30% considering the climate factor, and from 9.9% to 26.4% depending on soil features, including pH (Li et al. 2022). The study called for a more systematic collection of data to accurately assess the effectiveness of biostimulants. Notwithstanding, the precise mechanisms and signatures of biostimulants remain less well‐defined and understood at the metabolism and cellular levels (Baltazar et al. 2021; Martínez‐Lorente et al. 2024). Therefore, a particularly noteworthy challenge is the lack of clearly defined claims that guarantee the effectiveness of biostimulants. The European Committee for Standardization has proposed a requirement for providing robust evidence to substantiate the claimed effects of these products (CEN 2022). However, achieving this goal may require more than just demonstrating agronomic features. Identifying specific and common sub‐molecular signatures of biostimulants provides valuable insights into how these substances can be generalized or used across different crops. The application of advanced omics technologies, such as genomics, proteomics, and metabolomics, has significantly enhanced our understanding of how biostimulants modulate metabolic pathways (Nephali et al. 2020). Recent studies leveraging these technologies have increasingly focused on elucidating the precise biochemical and molecular mechanisms underlying biostimulant effects (Garg et al. 2024; Monterisi et al. 2024). The comprehensive nature of untargeted metabolomics has been used to highlight the shift in secondary metabolite signatures, particularly phenylpropanoids, flavonoids, and nitrogen‐containing compounds, rather than osmolytes and hormone profiles in plants exposed to different biostimulants (Salehi et al. 2025; Zhang et al. 2023). This deeper understanding enables more accurate predictions of biostimulant performance across various plant species and environmental conditions, thereby facilitating the optimization and effective application of these products. Despite the general effectiveness of biostimulants in enhancing plant growth and resilience across crops, most studies published to date have largely reported crop‐specific or trial‐related modes of action at the molecular level. Comparatively fewer studies have attempted to identify metabolic signatures associated with biostimulants using supervised statistical analyses. For example, a recent study used a supervised approach, AMOPLS, to identify the most discriminating metabolites associated with the effect of vermicompost application in Arabidopsis plants under salt stress (Salehi et al. 2025). Notably, it should be kept in mind that most of the available studies are limited to one biostimulant–crop combination, highlighting that the reported metabolic markers are often specific to that experimental context.

This limitation could be addressed by integrating omics data from a single experiment or from studies using different biostimulants and a range of plant species, allowing broader patterns to be identified. Leveraging machine learning approaches in this type of data integration offers a promising way to extract shared patterns across distinct datasets (García‐Pérez et al. 2025). Machine learning algorithms can analyze vast and complex datasets, uncovering hidden patterns and relationships that might not be apparent through traditional analytical methods (Rehman et al. 2025). By doing so, machine learning can assist in identifying common molecular signatures and predicting the efficacy of biostimulants across various conditions and plant species. This level of analytical resolution can support the development of standardized evaluation frameworks, improve the targeting of biostimulant applications, and optimize their formulation for specific crops and growing conditions. Such approaches may reduce variability in plant responses and improve the reproducibility of biostimulant performance across experiments. Integrating these analytical tools into biostimulant research, therefore, represents a practical step toward more evidence‐based and mechanistically informed agricultural practices.

This study aims to integrate the metabolomics datasets obtained from the application of six nonmicrobial biostimulants to three agriculturally important crops, tomato, cucumber, and pepper, grown under identical environmental conditions. The objective is to evaluate whether supervised statistical analyses combined with machine‐learning approaches can identify metabolic features associated with specific biostimulants that are consistently shared across these crops. Therefore, the investigation of the specific mode of action of the biostimulants is not the focus of the present study; the test products are commercial biostimulants already on the market and were chosen as representative products. Similarly, the test crops have been identified as representative horticultural crops where biostimulants are typically applied.

## 2 Materials and Methods

### 2.1 Experimental Design and Biostimulant Application

In this study, three widely used horticultural crops were selected: tomato ( *Solanum lycopersicum* L.), cucumber ( *Cucumis sativus* L.), and pepper ( *Capsicum annuum* L.). The experiment was conducted under tunnel conditions to simulate a controlled environment. A randomized block design (RBD) method was adopted, designating specific areas for each crop. The biostimulants used in this study were representative commercial formulations provided by Sofbey S.A., differing in their bioactive components: proline (Pro), melatonin (Mel), trehalose (Tre), organic matter (Org), seaweed extract (Swe), and the multi‐component O + Z. This latter formulation is composed of osmolytes and includes the cytokinin phytohormone zeatin. Each plant was exposed to six different biostimulant treatments plus a control, resulting in a total of 21 treatments. For each condition, four individual plants were used. The biostimulants were diluted in water to achieve application rates of 2 L/ha for Pro, 7 g/ha for Mel, 400 g/ha for Tre, 1.5 L/ha for Org, 1 L/ha for Swe, and 1500 g/ha for O + Z. The total spray volume for each biostimulant and the control treatment was 300 L/ha. According to the manufacturers' instructions, the biostimulants were foliar applied. Distilled water was used for the control plants.

The experiment, conducted from August to November, encountered natural suboptimal conditions due to ambient temperature variations in the field, which resulted from seasonal fluctuations rather than controlled, artificial conditions. The temperature and humidity data throughout the experiment are provided as Supporting Information (Figure S1). Biostimulants were applied to the plants at six different stages: (A) 7 days after transplanting (DAT); (B) at BBCH 59 (Biologische Bundesanstalt, Bundessortenamt and CHemical industry), during the formation of the first bunch; (C) at BBCH 59, during the formation of the second bunch; (D) 14 days after stage C; (E) 14 days after stage D; and (F) 14 days after stage E. Sampling for metabolomics analysis was conducted 3 days after the second biostimulant application. This coincided with the period during which the plants experienced the highest temperature range, beyond optimal conditions, from August to early September. For metabolomics, leaves were collected from the upper one‐third part of the plants and immediately frozen at −20°C until the extraction.

### 2.2 Extraction Procedure and UHPLC/QTOF‐MS Untargeted Metabolomics Profiling

The frozen leaves were ground using a mortar and pestle into a fine powder in liquid nitrogen. One gram of each pool was extracted with 10 mL of 80% (v/v) aqueous methanol acidified with 0.1% (v/v) formic acid, vortexed for 1 min, and then sonicated for 20 min. The extract was then centrifuged at 4°C for 15 min at 8000 × *g*, and the supernatants were syringe‐filtered through cellulose filters with a 0.22‐μm pore size (Salehi et al. 2025).

The extracts were then injected into ultra‐high performance liquid chromatography coupled with quadrupole time‐of‐flight mass spectrometry (UHPLC/QTOF‐MS). This was conducted with an Agilent 1290 series chromatograph and a G6550 mass spectrometer equipped with an electrospray ionization (ESI) source. The chromatographic conditions employed a Poroshell 120 PFP column (2.1 × 100 mm, 1.9‐μm particle size, Agilent). The mobile phase was a binary mixture comprising water (solvent A) and acetonitrile (solvent B), both acidified with 0.1% (v/v) formic acid. Elution was conducted using a constant gradient, decreasing from 94% to 6% A over 32 min, with a flow rate of 200 μL/min and an injection volume of 6 μL. The mass spectrometry acquisition employed positive polarity SCAN acquisition, covering a 100–1200 m/z range with a 30,000 FWHM resolution. The samples were injected twice following a randomized injection routine, together with blanks. Additionally, quality control (QC) samples were prepared for each crop by pooling equal aliquots (25 μL) from all individual samples. These QC samples were randomly injected throughout the sequence, specifically at the beginning and end of the batch, and every 10 analyzed samples. The QC samples were analyzed using data‐dependent MS/MS fragmentation (*N* = 9, collision energies of 10, 20, and 40 eV).

The raw data from UHPLC/QTOF‐MS were processed using the MS‐DIAL software (version 4.90) for automatic peak finding, LOWESS normalization, and annotation via spectral matching using the comprehensive Bioinformatics & Molecular Design Research Center Mass Spectral Library—Natural Products (BMDMS‐NP) and Fiehn/Vaniya natural product library (available at CompMS | MS‐DIAL (systemsomicslab.github.io)) for ESI‐MS/MS annotation. Features with a minimum peak height of 10,000 cps were retained, applying an accurate mass tolerance of 0.05 Da for MS peak centroiding and 0.1 Da for MS/MS, according to previously optimized alignment and identification parameters (Becchi et al. 2024). A Level 2 of confidence in annotation, with reference to the COSMOS Metabolomics Standard Initiative (i.e., putatively annotated compounds), was achieved in our experiments (Salek et al. 2013). The complete list of annotated compounds is available as Supporting Information (Tables S1–S3).

### 2.3 Statistics and Chemometrics

The data obtained from MS‐DIAL were initially processed using Mass Profiler Professional B.12.06 (Agilent Technologies). The raw abundances of the annotated features were normalized at the 75th percentile, transformed to log2 values, and baseline‐corrected to the median of all samples. After that, unsupervised hierarchical cluster analysis (HCA) was conducted using Euclidean distances and Ward's algorithm to naively explore patterns across treatments. Furthermore, a supervised analysis of variance (ANOVA) multiblock orthogonal partial least squares (AMOPLS) analysis was performed using R software (v. 4.2.3) with the “rAMOPLS” package (v. 0.2) (Boccard et al. 2019). This analysis was used to determine the statistical significance of the factors genotype, treatment, and their interaction (genotype * treatment) within our dataset. The model was built by optimizing the lowest number of statistically significant orthogonal components (To; *a* = 0.01), while retaining the predictive components (Tp) that maximized the explained variability associated with each factor. The quality of the model was evaluated using two parameters: relative sum of squares (RSS), which represents the proportion of variability explained by each factor, where higher RSS value indicates a greater contribution to overall variability; the residual structure ratio (RSR), which indicates the contribution of each factor relative to the unexplained residual variance, where higher RSR value reflects the higher contribution of a factor on the overall variability; and *p*‐value, which represents the statistical significance of each factor on the overall variability, setting a significance threshold (*α* = 0.01). Furthermore, 100 permutations and a 10‐step subsampling procedure combined with three‐step parallelization were carried out to account for variability in sample size across groups resulting from the unbalanced experimental design. The variables associated with discrimination for each factor were identified and represented by variable importance in projection (VIP^2^) markers and their corresponding VIP scores. Boruta feature confirmation was further performed by the “Boruta” R package, based on a random forest algorithm to identify and confirm the most relevant features related to biostimulant treatment (Kursa and Rudnicki 2010). Model robustness was assessed by performing an iterative removal of randomized replicates within each experimental group, observing no significant changes in the overall outcome of the integrative models.

### 2.4 Machine Learning‐Assisted Classification

The output datasets from metabolomics of three crops were jointly analyzed using the DIABLO (Data Integration Analysis for Biomarker discovery using Latent variable approaches for Omics studies) framework, accessible through the “mixOmics” package within R software (v. 6.22) (Rohart et al. 2017). Initially, four independent sparse Partial Least Squares sPLS models were applied to estimate the correlation between metabolomics datasets across the crops. Subsequently, DIABLO modelling, based on multiblock sPLS discriminant analysis (sPLS‐DA), was performed to evaluate the identified correlations at the component level across the different biostimulants. The DIABLO model was optimized by adjusting the tuning function. This involved determining the optimal number of components, selecting those with the lowest overall balanced error rate, and utilizing the centroid distance for maximum accuracy. Additionally, the number of variables selected for each dataset was determined through repeated 10‐fold stratified cross‐validation. Model validation was performed through Receiver Operating Characteristic (ROC) curve analysis, setting a discrimination threshold of 0.9 for the integrative models performed for each species.

## 3 Results and Discussion

Despite the growing use of biostimulants and the availability of numerous studies investigating their effects on plants, many scientists point out a lack of consistent scientific evaluation (Garg et al. 2024). This is likely due to crop‐specific responses, where a given biostimulant may induce beneficial effects in one crop but not in another (Ruzzi et al. 2024; Zulfiqar et al. 2024). Large datasets from omics approaches have been used to uncover the mode of action of biostimulants (Baghdadi et al. 2022; Sudiro et al. 2022). In this study, untargeted metabolomics led to the putative annotation of 1203, 1647, and 1387 metabolites in cucumber, pepper, and tomato, respectively. The combined metabolomics datasets from the three crops were considered for further statistical analyses. Therefore, the strategy used in this study was not to analyse and interpret the metabolomics results of each crop and each biostimulant individually, as many previous studies have done, but instead to integrate all data and examine whether multivariate statistical analyses combined with machine‐learning tools assist in identifying single small molecules across all three crops, despite the highly crop‐specific nature of metabolomics. Such generalized outcomes are challenging to identify but may have broader interest than crop‐specific effects.

### 3.1 Supervised Statistical Analyses Revealed the Dominance Effect of the “Genotype” Factor

**FIGURE 1:**
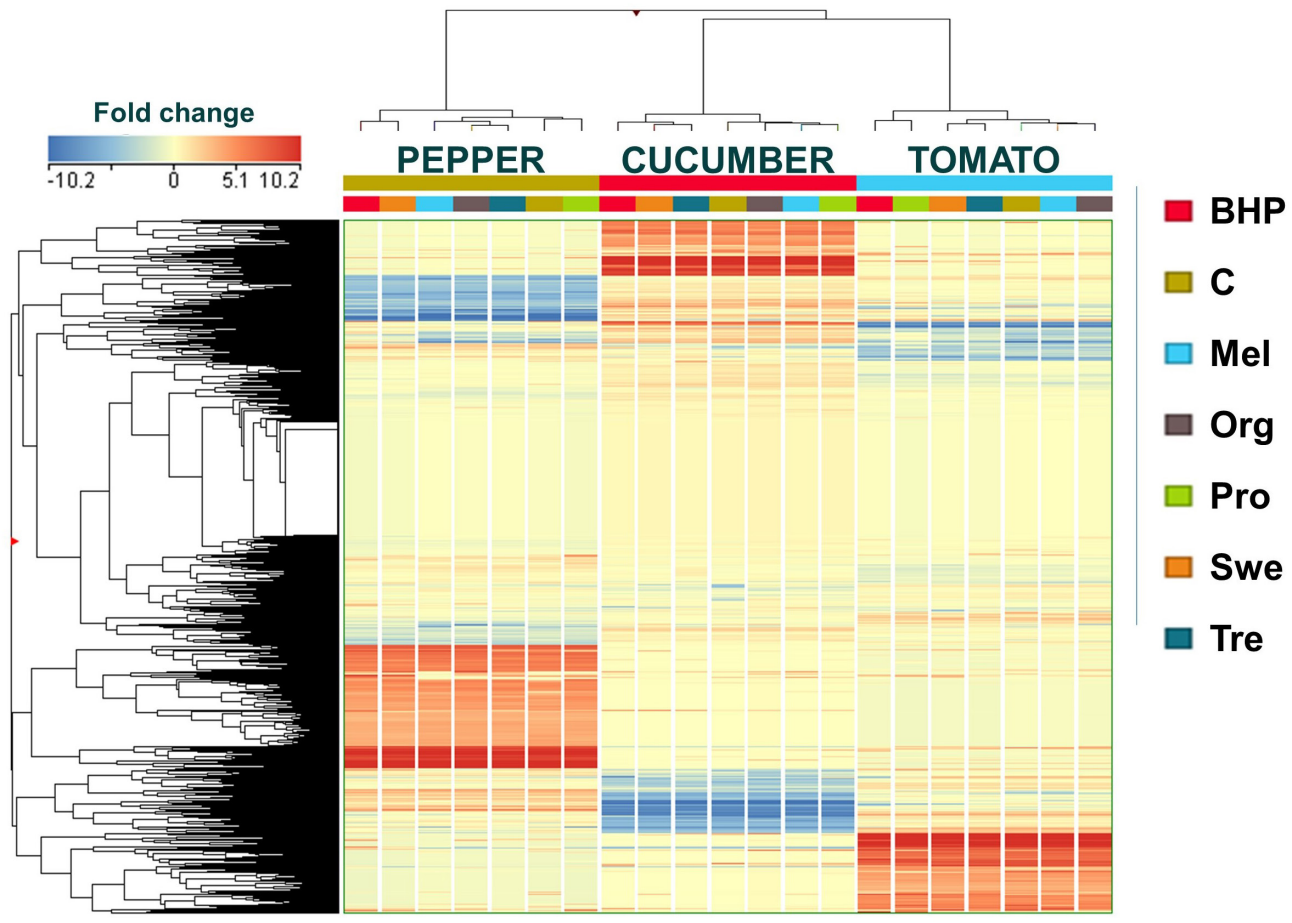
Heatmap generated from unsupervised cluster analysis (HCA) of the metabolomics profiles of cucumber, pepper, and tomato, each tested with various biostimulants under nonoptimal naturally high temperature conditions.

Metabolomics studies previously have revealed that the metabolic profiles of plants are highly specific to each species, even under similar conditions (Bulgari et al. 2019; Marr et al. 2021). In our study, unsupervised HCA was initially performed using the average fold‐change variations across all samples, considering the factors “genotype” and “treatment” (Figure 1). This analysis aimed to naively display patterns within the dataset, highlighting similarities and differences among the crops and the treatments, and pointing out the hierarchy of the factors involved. The three genotypes formed three primary clusters, each reflecting unique metabolic profiles for the three crops, as expected. However, no uniform clustering pattern was observed for the treatment factor across crops, as the same biostimulant was grouped differently depending on the species. This crop‐dependent variation suggests that biostimulant application does not induce a conserved metabolic change but rather interacts with the internal metabolic framework of each crop, resulting in the divergent clustering pattern (Kursa and Rudnicki 2010; Rinaudo et al. 2016), which showed that biostimulant effectiveness is closely linked to genotype and metabolic background. Delving deeper into the clustering pattern on each crop revealed that the multi‐component biostimulant containing osmolytes and zeatin (O + Z) consistently shifted the metabolic profile, as it clustered far from control plants in all three crops. In pepper, O + Z clusters with the Swe treatment, while in cucumber, it clusters with Swe and Tre, and in tomato, it aligns with Pro. In contrast, the Mel and Org treatments cluster with control plants across all crops, usually forming a single subcluster. This latter clustering pattern suggests that Mel and Org treatments induced a milder modulation of the metabolic profile from control plants.

**FIGURE 2:**
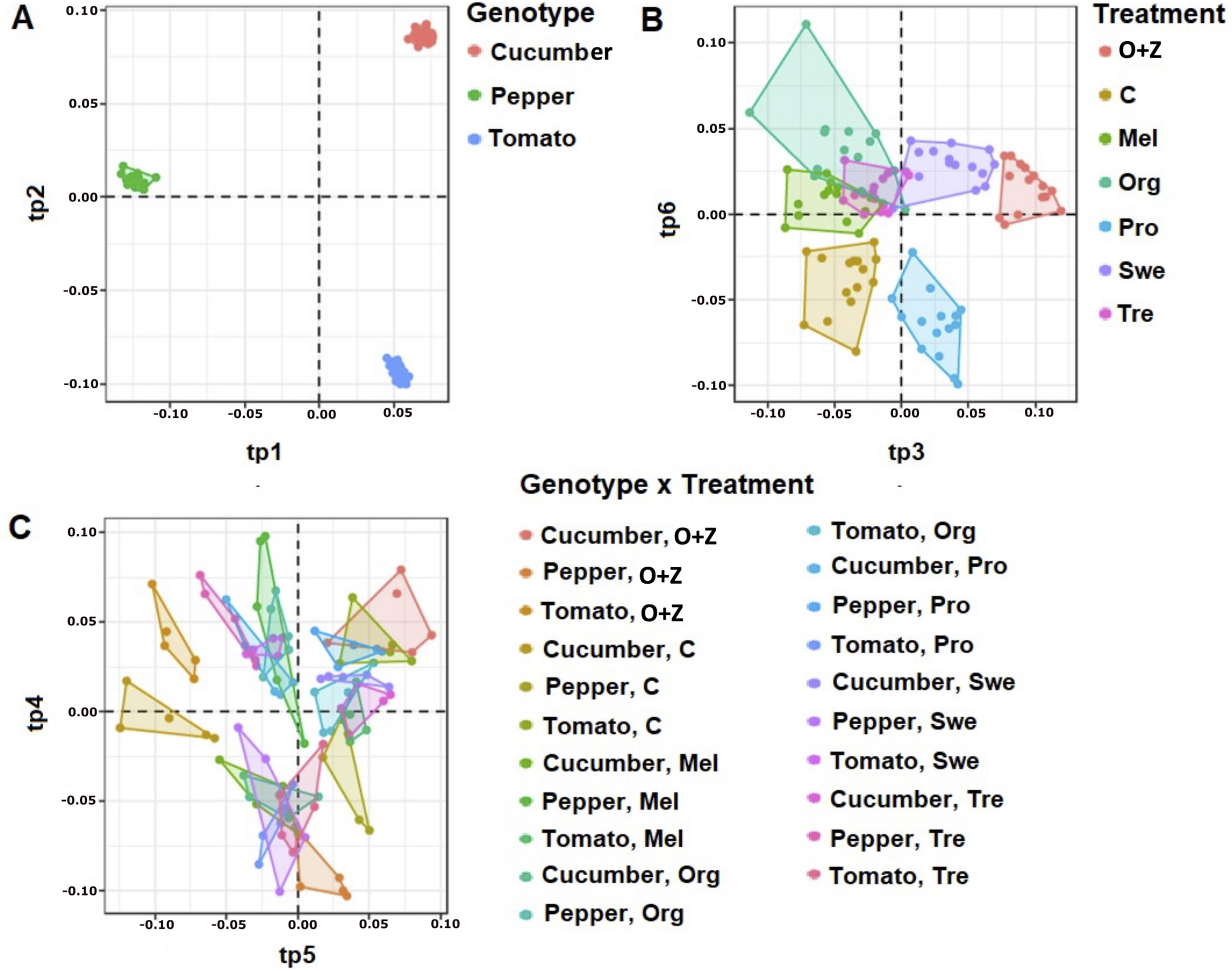
Supervised AMOPLS analysis score plots based on the metabolomics profile of three crops treated with different biostimulants under nonoptimal naturally high temperature conditions, highlighting three key discriminant factors: Genotype (A), Treatment (B), and Genotype × Treatment (C). Mel: melatonin; O + Z: multi‐component biostimulant (osmolytes and zeatin); Org: organic matter; Pro: proline. tp 1–6 represent predictive components.

Subsequently, given the dominance effect of “genotype” as shown by HCA, a supervised multivariate statistical analysis utilizing ANOVA‐multiblock orthogonal partial least squares (AMOPLS) was performed to elucidate precisely the influence of each factor. The model, trained by removing one replicate from each experimental group, did not alter the outcome of the DIABLO integrative analysis (Supporting Information S1). Given the robustness of modelling, the score plots for each factor, that is, genotype, treatment, and the interaction of genotype × treatment, have been exported and are presented in Figure 2. The three factors examined in this study were found to have a statistically significant impact on the metabolic profile (*R* ^2^ *Y p*‐value = 0.01). In parallel with HCA, AMOPLS, by considering the RSS criteria (i.e., the reliability of the factors involved), revealed that the highest variation is attributed to “genotype” at 68.2%. In comparison, “treatment” and the “genotype × treatment” interaction accounted for 3.5% and 5.6%, respectively. The remaining 22.6% pertains to “residuals,” representing the portion of data variability not explained by the factors considered. The AMOPLS models revealed a clear separation of the three crops across components tp1 and tp2, as already evident from HCA. In particular, pepper was separated from the other two crops along the tp1 component. Regarding the “treatment” factor, it can be inferred that O + Z had the most significant discriminating effect on the model, followed by the Swe, while Pro exhibited similarities with the control plants. The same discriminating effect of O + Z was observed when considering the “genotype × treatment” factor, as expected in a genotype‐dependent manner.

### 3.2 Identification of the Most Discriminant Features Related to the “Treatment” Factor Validated by AMOPLS and Boruta Analyses

**FIGURE 3:**
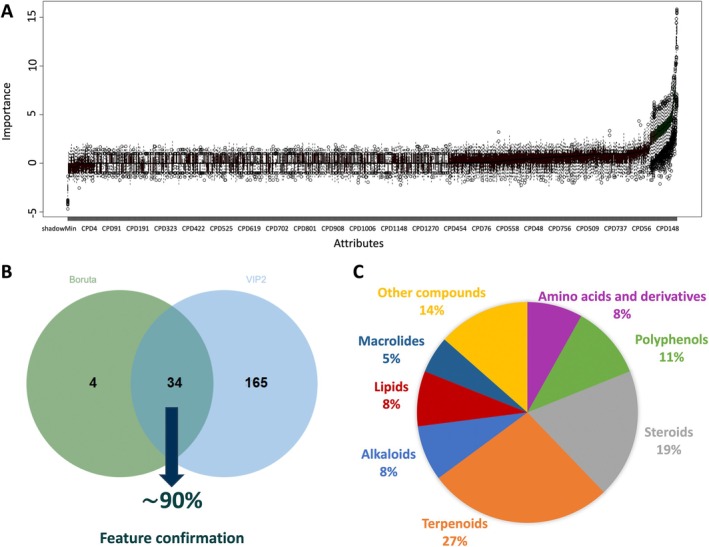
Boruta feature selection identifying key features associated with biostimulant treatments (A). Venn diagram showing the significant overlap of selected metabolite features between Boruta and AMOPLS analysis (B). Pie chart representing the metabolite classes of the confirmed features. Mel: melatonin; O + Z: multi‐component biostimulant (osmolytes and zeatin); Org: organic matter; Pro: proline.

Considering that this study aimed to examine a novel approach to identifying generalized signatures of biostimulants across various crops, we specifically focused on the features associated with the “treatment” factor. From the supervised AMOPLS, a total of 199 VIP^2^ features (Table S4) with the highest scores were extracted for further analyses. The complete list of VIP^2^ features identified for each factor is provided in the Supporting Information (Table S4). When dealing with large datasets, especially in metabolomics, researchers encounter hundreds of metabolites and should consider various aspects, including cheminformatics, bioinformatics, and statistics (Cambiaghi et al. 2017). While the identification of all metabolites provides a complete view, selecting the most promising candidates may be used to identify the most robust signatures that contribute significantly to model performance (Kursa and Rudnicki 2010; Rinaudo et al. 2016). To this aim, a Boruta feature selection analysis based on Random Forest‐derived variable importance was applied to discriminate features specifically relevant to the “treatment” factor. The analysis led to the selection of 38 metabolites that contributed to discriminating between samples by biostimulant treatment (Figure 3A). This parallel approach ensured the robustness of our findings by validating the features identified by AMOPLS through an independent, data‐driven method, enhancing the reliability of the relevant signatures (Labory et al. 2024). The Venn diagram comparing the features identified by AMOPLS and Boruta highlighted an approximate 90% overlap in the confirmed features (Figure 3B). This observation suggests that Boruta, while effective in highlighting important features, can be computationally demanding, especially when working with large datasets. This increased computational load arises from the method's requirement to perform multiple iterations and to repeatedly train the underlying model. Nevertheless, Boruta offers a clear and systematic approach to evaluating feature importance by ranking features according to their relevance (Alsahaf et al. 2022). This algorithm has been used in different research fields, including medicine and food science, where it has been reported to improve the accuracy of predictive models and facilitate the interpretation of complex datasets (Bain et al. 2025; Basu et al. 2023; García‐Pérez et al. 2024; Iranzad and Liu 2025; Marcos‐Zambrano et al. 2021). In this study, the features identified for discriminating among various biostimulants across all three crops primarily included amino acid derivatives, such as betaine; polyphenols, comprising 11% of the features and represented by (*E*)‐*N*‐caffeoylputrescine; terpenes, accounting for 27%, which encompassed di‐, tri‐, and sesquiterpenoids; steroids, constituting 19%; and alkaloids (Figure 3C). These classes of metabolites have been widely reported as stress‐related compounds that contribute to plant resilience under various abiotic stress conditions (Chen et al. 2022).

### 3.3 Machine‐Learning Approaches Identified the Key Markers Shared Among the Three Crops for the O + Z Biostimulant

**FIGURE 4:**
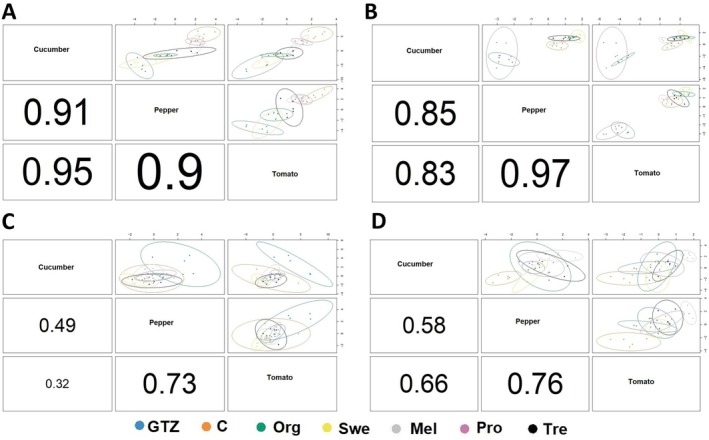
DIABLO‐based data integration models for the metabolomics of the three crops (pepper, cucumber, and tomato) treated with different biostimulants under nonoptimal naturally high temperature conditions. Samples are represented based on the specified components (ncomp = 4) for each crop's data set, with samples colored according to biostimulant type. A, B, C, and D represent the correlation matrix comparing the three data blocks (i.e., pepper, cucumber, and tomato) involved in the first, second, third, and fourth components, respectively. The axes indicate the projection of each sample onto the latent components extracted by the sPLS algorithm. The numbers on the plot axes represent the latent variable scores. Each sample receives a score indicating its position along that component. Samples clustering together have similar metabolomic profiles across integrated blocks. Samples far from zero express the pattern captured by that component, while samples on opposite sides of zero have contrasting metabolomic signatures for that component. As shown in the plot, component 1 captures the most discrimination. Mel: melatonin; O + Z: multi‐component biostimulant (osmolytes and zeatin); Org: organic matter; Pro: proline.

**FIGURE 5:**
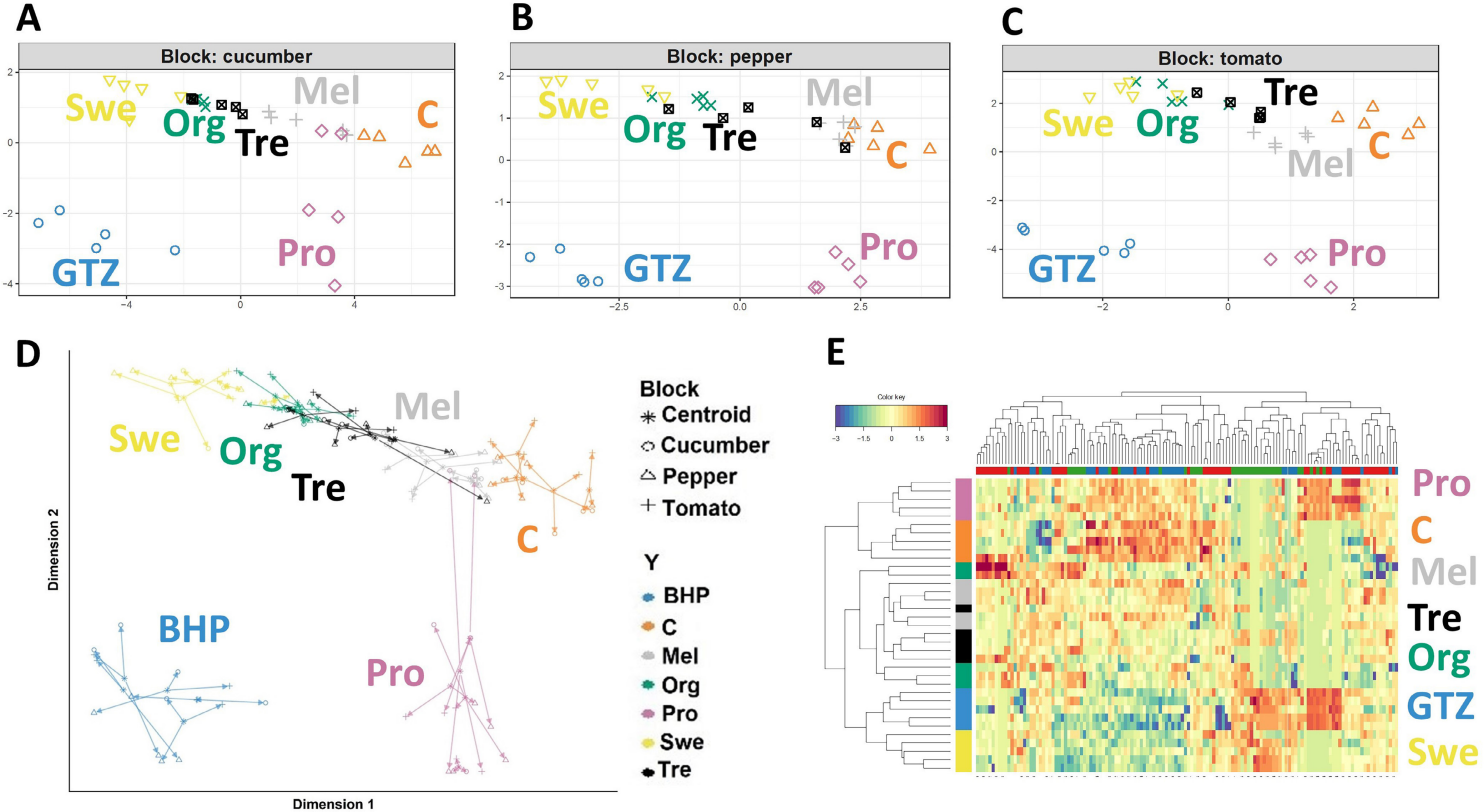
Block contributions for the metabolomic profile of cucumber (A), pepper (B), and tomato (C). The samples are plotted according to their scores on the four components for each metabolic data set. Arrow plot from multiblock sPLS‐DA performed on the metabolic profile of three crops (D). The samples are projected into the space spanned by the four components for each data set, then overlaid across all three metabolic data sets. The start and tip of the arrow represent the centroid between all datasets for a given sample and the location of the same sample in each block. Heatmap correlation plot of the most discriminant features selected from metabolomics datasets (E). The biostimulant type is represented in rows, and the selected metabolites are represented in columns. The colors represent the biostimulant type. Mel: melatonin; O + Z: multi‐component biostimulant (osmolytes and zeatin); Org: organic matter; Pro: proline.

**FIGURE 6:**
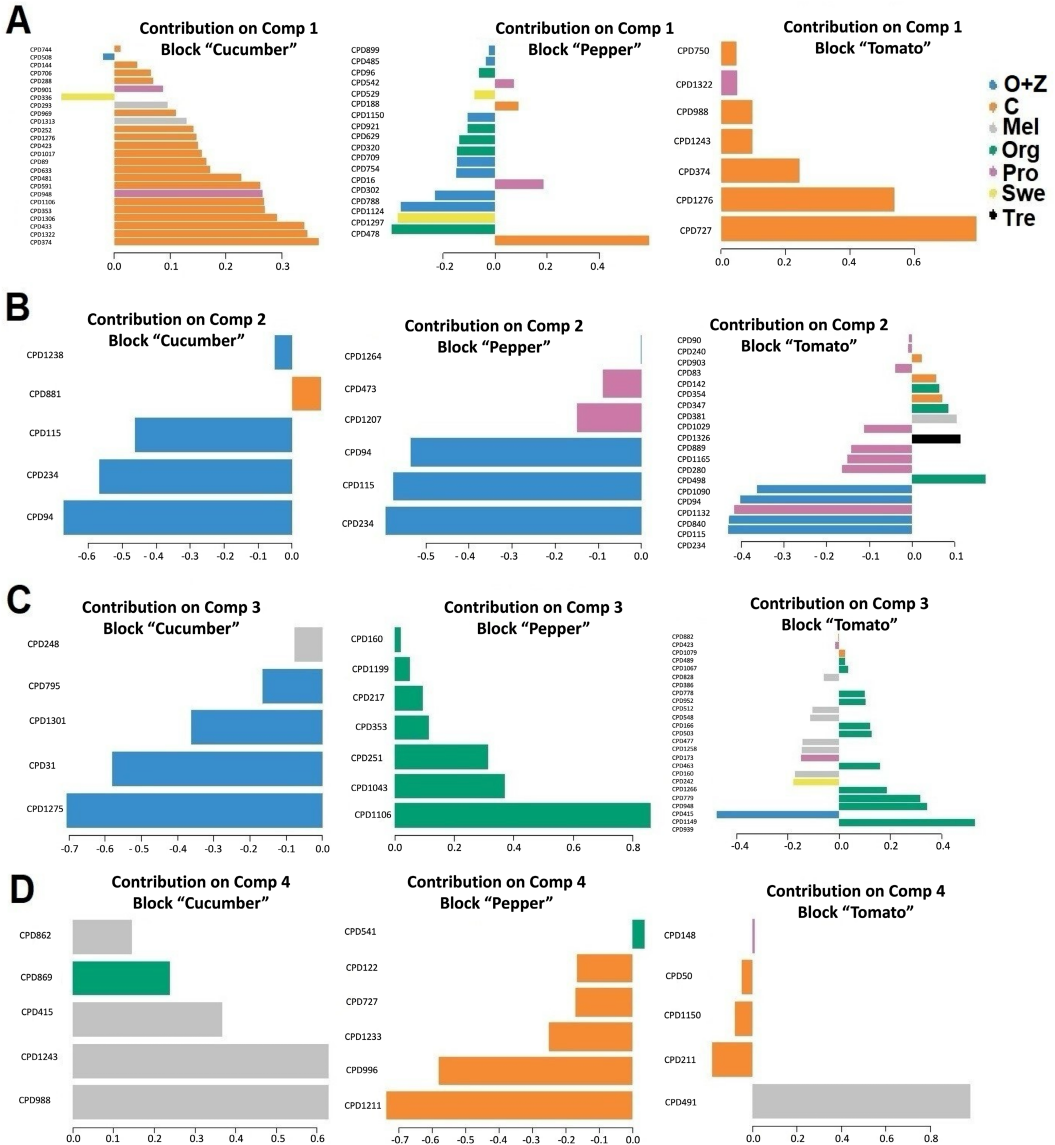
Loading plots of each metabolite selected, having the maximal discrimination ability on the first (A), second (B), third (C), and fourth (D) components in each crop. The most significant metabolites are ordered from bottom to top. Colors indicate the biostimulants type for which the median fold‐change value is the highest for each metabolite. Mel: melatonin; O + Z: multi‐component biostimulant (osmolytes and zeatin); Org: organic matter; Pro: proline.

Following the use of Bourta and AMOPLS to extract features explicitly related to the “treatment” factor, we utilized DIABLO. DIABLO represents a novel multivariate integrative classification method developed to build a predictive model for prediction on new samples. It is based on Projection to Latent Structures (PLS), which allows powerful data visualization (Munk et al. 2024). This method is highly versatile in the type of experimental design it can handle (Singh et al. 2019). First, sPLS models were built based on the four components for each crop, resulting in highly significant correlation coefficients between pairs: 0.91 for cucumber and pepper, 0.95 for cucumber and tomato, and 0.90 for pepper and tomato (Figure 4). These high correlation coefficients suggest that the features important for one crop are similarly crucial for the others, indicating a consistent pattern across these crops. Such correlations further suggest that results obtained from one crop could, to some extent, be informative for the others. Subsequently, the sPLS‐DA analysis was performed to show the impact of biostimulants on the metabolic profiles of the three crops (Figure 5A–C). In all models, the first predictive component clearly separated O + Z from Pro, while the second predictive component separated O + Z and Pro from the other biostimulants. Comparison of the sPLS models across crops showed a consistent trend in the separation of biostimulants, which suggests similar metabolic modulation by each biostimulant regardless of crop type (Figure 5D). This separation was consistent with HCA, which showed two primary clusters, with Pro and Control grouped in one cluster, and the remaining treatments in another. Deepening in the latter cluster revealed two subclusters: one containing Swe and O + Z and the other comprising Mel, Org, and Tre (Figure 5E). The supervised DIABLO analysis provided metabolites associated with the separation observed among the biostimulants. Here, the analysis revealed that the four principal predictive components are essential for identifying the key markers that govern the model. The markers associated with each block for the four principal components are illustrated in Figure 6. Considering the first component (Figure 6A), the features were primarily attributed to distinguishing the control group, particularly in cucumber and tomato, while they were linked to the O + Z and Mel treatments in pepper. For the second component (Figure 6B), the features were associated with the differentiation of the O + Z treatment across all three crops. The third component was predominantly related to O + Z in cucumber and Org in pepper (Figure 6C). The fourth component was attributed to the control and Mel treatments (Figure 6D). For tomato and cucumber, only O + Z and Mel caused a discriminative metabolic signature, revealing another common functional trait ascribed to these biostimulants; however, in the case of pepper, all biostimulants (except Tre) led to a discriminant output (Figure S2). This suggests that pepper is more prone to metabolic reprogramming under biostimulant application.

**FIGURE 7:**
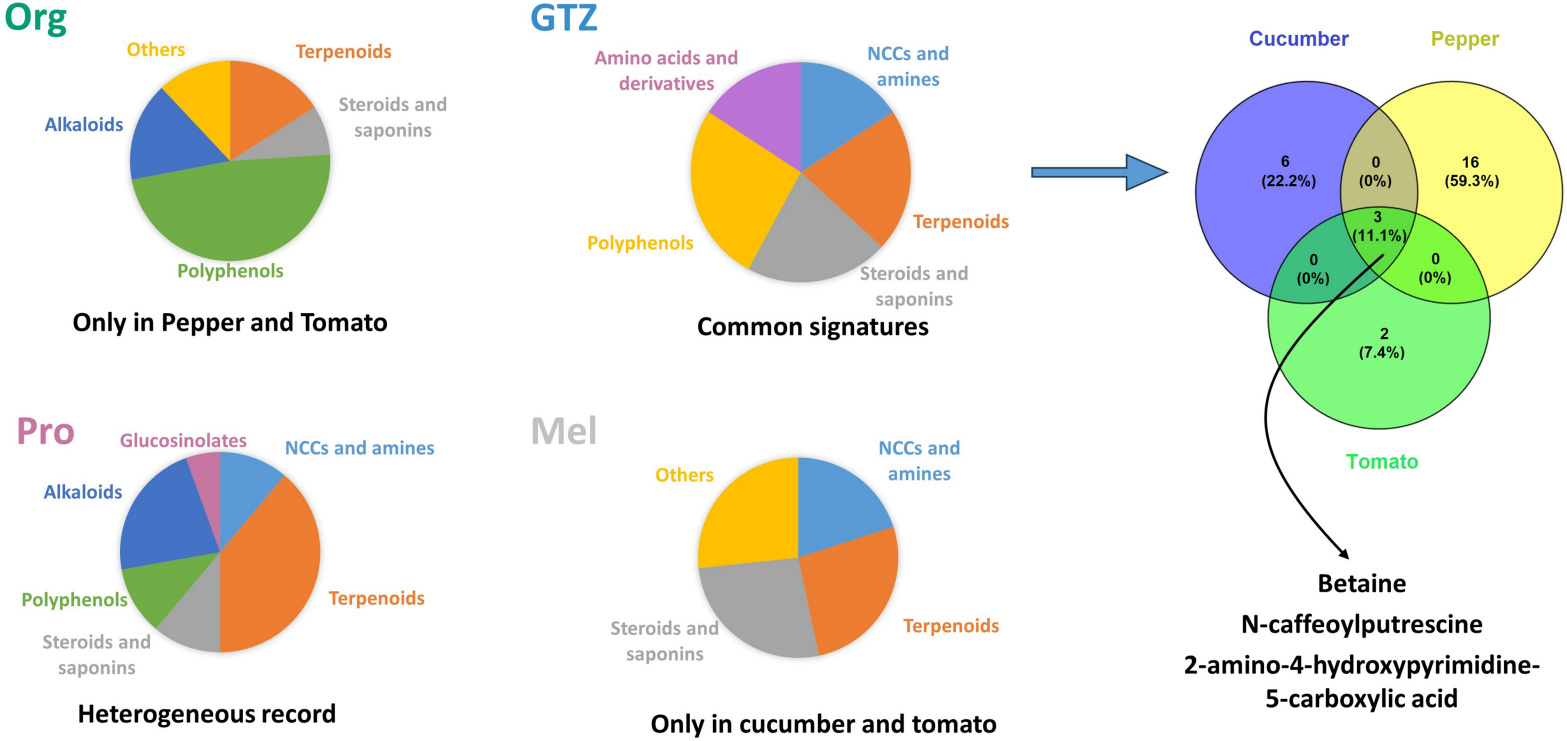
Pie chart representing the most relevant classes of selected metabolites for four representative biostimulants (right). Venn diagram showing the overlap of three identified biomarkers for O + Z across three crops. Abbreviations: Mel: melatonin; NCCs: nitrogen‐containing compounds; O + Z: multi‐component biostimulant (osmolytes and zeatin); Org: organic matter; Pro: proline.

Discriminant features associated with each biostimulant differed among treatments (Figure 7). Under the Org treatment, most discriminant features were detected in pepper and tomato, with polyphenols accounting for 48% of compounds, followed by terpenoids and alkaloids. A metabolite annotated as a steroidal saponin was detected in both pepper and tomato plants. In contrast, the Pro treatment showed discriminant features across all crops, with terpenoids and alkaloids being the most abundant classes, together with several metabolites related to polyphenols and glucosinolates. Under the Mel treatment, the discriminant features were mainly represented by terpenoids and steroids, together with several NCCs and amines. A similar feature classification was observed for the O + Z treatment. Among the features, three metabolites, including betaine, *N*‐caffeoylputrescine, and 2‐amino‐4‐hydroxypyrimidine‐5‐carboxylic acid, were detected in all crops. Betaine is a well‐known osmoprotectant involved in plant responses to abiotic stress via the maintenance of cellular water balance and protection against dehydration. In addition, betaine contributes to the stabilization of cellular structures and enzyme activity (Quan et al. 2023; Saeed et al. 2017; Zulfiqar et al. 2022). N‐caffeoylputrescine is a conjugated polyamine that has been reported to participate in plant stress responses. Polyamines contribute to antioxidant defense and the mitigation of oxidative stress through free radical scavenging activity, and the regulation of stress‐related pathways (Jing et al. 2020; Upadhyay et al. 2020). 2‐Amino‐4‐Hydroxypyrimidine‐5‐Carboxylic acid belongs to pyrimidine‐derived compounds, which have been reported to influence the synthesis of secondary metabolites via modulation of pyrimidine metabolism towards increased availability of uracil for secondary metabolism (Brown and Turan 1995). The results of this study revealed that O + Z biostimulant, which is composed of three components (glycine‐betaine, trehalose, and zeatin), differentially modulates the metabolic profile of crops compared to other biostimulants. Biostimulants with only one component are likely to affect only a few metabolic pathways or physiological processes, which may reduce their overall performance. For example, proline is an osmoprotectant that helps to stabilize macromolecules, such as proteins, and to maintain the integrity of the membranes. In contrast, multi‐component biostimulants contain multiple active compounds that can be involved in more than one metabolic or physiological process in plants. Previous studies have reported that formulations composed of several components may perform differently from single‐component products, particularly under stress conditions, as more than one pathway can be affected at the same time (Asif et al. 2023; Ricci et al. 2019). For instance, a biostimulant composed of flavonoids and phenolic acids, including protocatechuic acid, quercetin, chlorogenic acid, coumaroyl quinic acid, and gentistic acid, improved photosynthesis and increased tuber yield in three potato varieties (Salvage et al. 2024). Another study reported that the combined application of trehalose and salicylic acid had a greater impact on mitigating drought‐induced oxidative stress in sweet basil than each component individually (Zulfiqar et al. 2021). In this study, the combination of glycine‐betaine (osmoprotectant), trehalose (a sugar), and zeatin (a hormone) may have modulated several metabolic pathways; as a result, three common compounds were detected from DIABLO analysis.

Traditionally, studies that evaluate the effect of biostimulants on plants have mainly reported crop‐specific responses (Kisvarga et al. 2022; Parađiković et al. 2019; Zulfiqar et al. 2024). Despite confirming crop‐dependent metabolomic profiles, however, the integrated data analysis in this study revealed common metabolic signatures shared among the three crops, suggesting that the effects of biostimulants may be more universally applicable than previously thought. Integrating multiple omics layers can reveal novel insights and a deeper understanding that single‐layer analyses cannot achieve. This study indicated the feasibility of applying this approach to three metabolomics datasets to identify markers associated with various biostimulants across the three crops.

## 4 Conclusions

This study provides novel insights into how large metabolomics datasets can be handled to go beyond the crop‐specific mode of action of biostimulants. Unlike previous approaches, metabolomic datasets from three different crops were jointly analyzed. Despite the small sample size, clear themes emerged from combining high‐resolution metabolomics with advanced multivariate data integration approaches. The Venn diagram, which was applied to the machine‐learning‐derived most discriminant features, indicated that three compounds were consistently present in all three crops and were associated with the multi‐component biostimulant O + Z. The identified metabolites included betaine, *N*‐caffeoylputrescine, and 2‐amino‐4‐hydroxypyrimidine‐5‐carboxylic acid, which could potentially be involved in defense responses when plants face detrimental conditions. The convergence of these metabolites may underlie a conserved and reproducible mode of action of O + Z, clearly distinguishing it from single‐component biostimulants, which typically showed crop‐specific metabolic signatures. Indeed, this consistent behavior can likely be attributed to the composition of the O + Z biostimulant, especially due to the presence of the phytohormone zeatin.

Generally, our research showed that integrating omics data offers advances beyond evaluating crops individually and offers avenues to reduce complexity and inconsistency in biostimulant investigations. Therefore, the analytical and chemometric procedures performed in this study could potentially be used to predict and fine‐tune the efficiency of a given biostimulant across different plants, thus providing guidelines for developing new products for specific targets. Still, to deeply validate this approach, future studies are necessary to be performed on a broader range of crop plants and with a larger sample size.

## Author Contributions

**Hajar Salehi:** investigation, formal analysis, data curation, software, visualization, writing – original draft. **Pascual García‐Pérez:** data curation, software, visualization. **Luigi Lucini:** conceptualization, supervision, writing – review, and editing. All authors contributed to the article and approved the submitted version.

## Funding

The authors have nothing to report.

## Supporting information


**Table S1:** Metabolomics dataset (annotated with MS‐DIAL) of cucumber olant treated with different biostimulants underheat stress conditions.
**Table S2:** Metabolomics dataset (annotated with MS‐DIAL) of pepper olant treated with different biostimulants underheat stress conditions.
**Table S3:** Metabolomics dataset (annotated with MS‐DIAL) of tomato olant treated with different biostimulants underheat stress conditions.
**Table S4:** VIP^2^ markers name and values of the most significant contribution to the Genotype, Treatment, and Genotype × Treatment factors in the rAMOPLS model in leaf tissue.
**Table S5:** The identified metabolites based on four components in three crops.
**Figure S1:** Temperature and humidity data from the open‐field experiment.
**Figure S2:** Receiver Operating Characteristic (ROC) curve for the validation of the discrimination potential of the integrative DIABLO models performed for each species subjected to different biostimulant treatments. From top to bottom, validation of DIABLO model established for tomato, cucumber, and pepper.
**Figure S3:** Robustness of the AMOPLS model (leave one out approach: model output is on the left, model robustness on the right).

## Data Availability

The metabolomic data for *Solanum lycopersicum* L. (Study ID ST004754), *Capsicum annuum* L. (Study ID ST004753), and *Cucumis sativus* L. (Study ID ST004752) are available on Metabolomics Workbench (https://www.metabolomicsworkbench.org; Sud et al. 2016; work supported by NIH grant U2C‐DK119886 and OT2‐OD030544 grants). The data can be accessed directly via its Project https://doi.org/10.21228/M8K264 (https://dev.metabolomicsworkbench.org:22222/data/DRCCMetadata.php?Mode=Project&ProjectID=PR003018&Access=BetZ9934).
